# A tale of 2 torsades: How to approach a patient with torsades de pointes and distinguish between classical and pseudo–torsades de pointes

**DOI:** 10.1016/j.hrcr.2022.03.007

**Published:** 2022-04-15

**Authors:** Melvin Scheinman, T. Jared Bunch, Mohita Singh

**Affiliations:** ∗ Division of Cardiology, Section on Electrophysiology, University of California San Francisco, San Francisco, California; † Division of Cardiology, Department of Internal Medicine, University of Utah, Utah; ‡ Division of Cardiology, Department of Internal Medicine, University of Texas Southwestern Medical Center, Dallas, Texas

**Figure 1:**
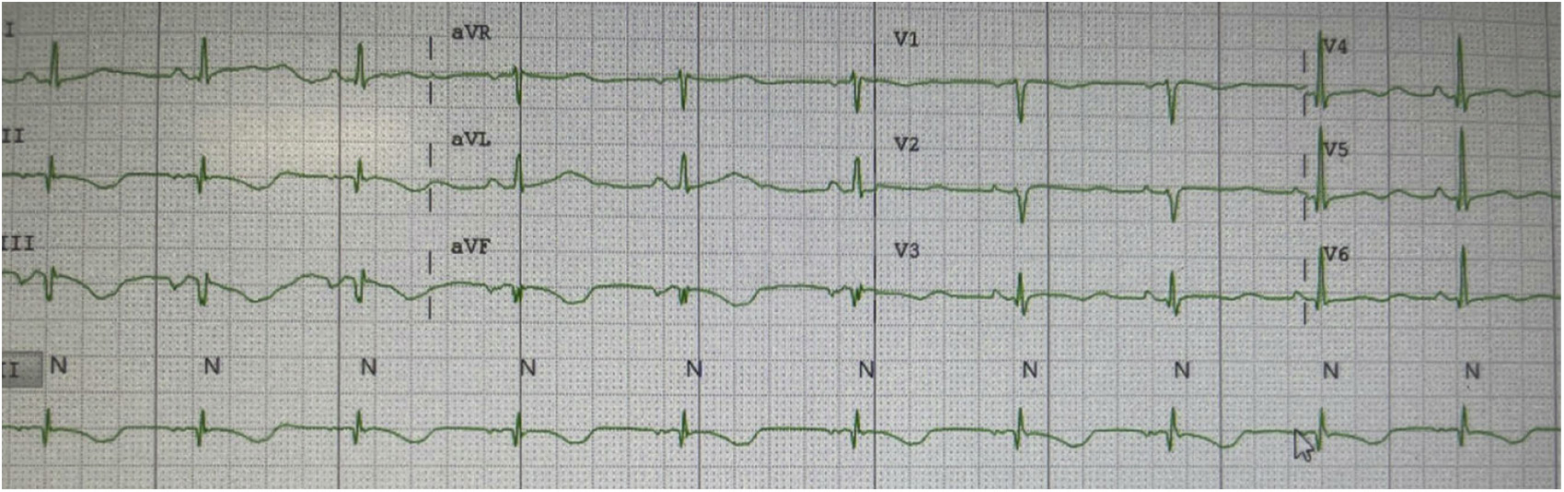
Baseline ECG for patient 1 notable for markedly prolonged QTc.

**Figure 2:**
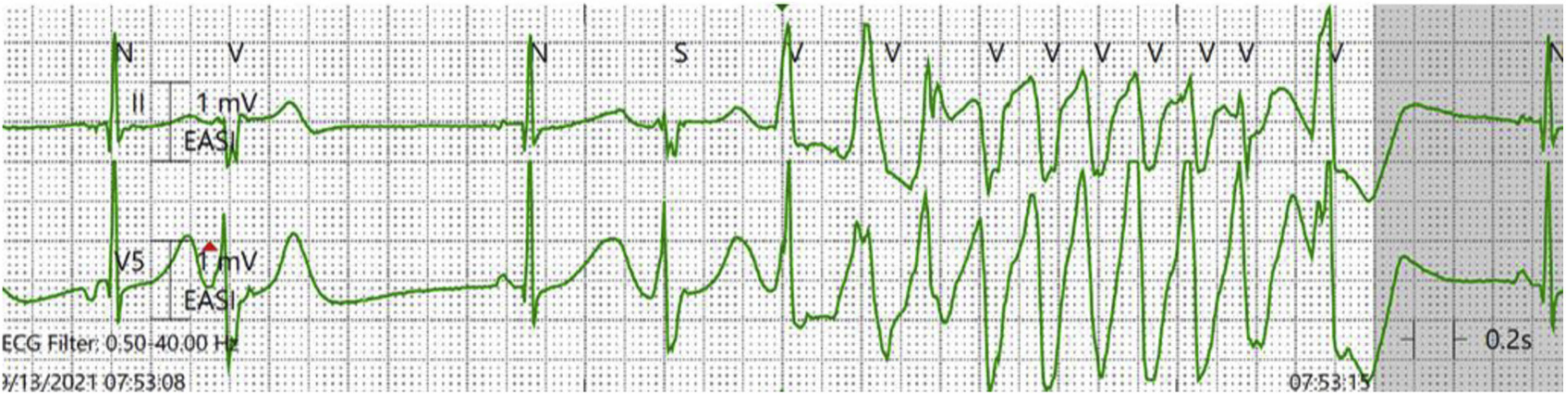
Initiation of torsades de pointes by a short-long-short episode in patient 1.

*Dr Scheinman:* The purpose of this essay is to review the approach to evaluation of the patient who presents with an electrocardiogram (ECG) showing rapid polymorphic ventricular tachycardia (PVT). We begin by presenting 2 patients with rapid polymorphic arrhythmias suggestive of torsades de pointes (TdP). The first case involves a 45-year-old woman who presents with cyclical emesis, palpitations, and near syncope. Her electrolyte panel shows a serum potassium of 3.5 mEq/L and serum magnesium of 2.2 mg/dL. The baseline ECG shows a markedly prolonged QTc (Figure 1) and a short-long-short episode that provokes an episode of TdP (Figure 2). She was treated with isoproterenol as well as replacement of potassium and magnesium, and the QTc normalized and the arrhythmia abated.

*Dr Bunch:* Dr Singh, when you see a patient who presents with PVT, what are the most important reversible causes to consider?

*Dr Singh:* The most common reversible cause to consider is the presence of electrolyte derangements, specifically hypokalemia, hypocalcemia, and hypomagnesemia, as these predispose patients to TdP and are easily reversible. Other reversible causes include presence of hypothermia and bradycardia. A thorough review and cessation of medications that can predispose to QT prolongation, especially diuretics, is usually helpful.

**Figure 3:**
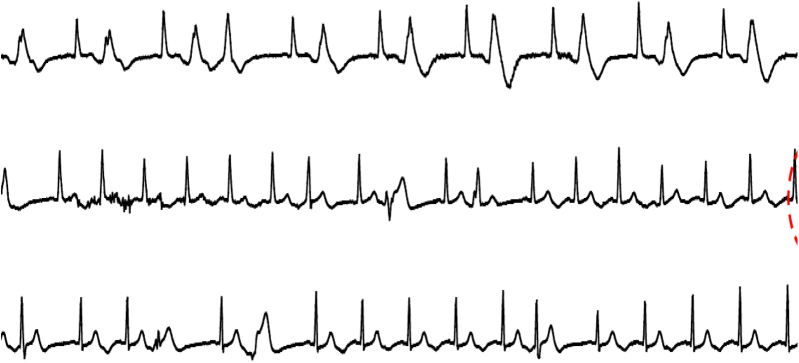
Baseline ECG for patient 2 notable for frequent PVCs and a prolonged QTc of 480 ms.

**Figure 4:**
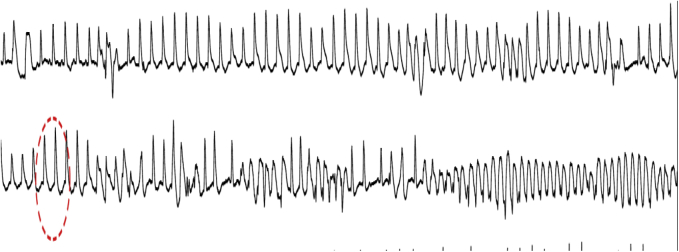
Patient 2 with polymorphic VT.

The second patient presented with palpitations and chest pain. He was taking citalopram and ketoconazole. The baseline ECG showed a QTc of 486 ms and frequent premature ventricular complexes (PVCs) (Figure 3) and bursts of PVT (Figure 4). Coronary angiography showed severe triple vessel disease and he underwent urgent revascularization, with no further sustained arrhythmias despite persistent PVCs.

*Dr Bunch:* Dr Singh, in patients with PVC-triggered PVT, is the timing of triggering after depolarizations important, and does this help guide antiarrhythmic drug therapy choices?

*Dr Singh:* In long QT syndrome (LQTS), late coupling PVCs lead to early afterdepolarization, which can then trigger TdP. Usually in congenital LQTS type I and II, we can expect late PVCs at intervals of >400 or even 500 ms, whereas the coupling interval triggering PVT in Brugada syndrome, idiopathic ventricular tachycardia (VT), and coronary artery disease is usually described as 302 ± 52 ms in idiopathic VT, 388 ± 28 ms in Brugada syndrome, and 364 ± 36 in patients with coronary artery disease.^1^ So in general in LQTS we can expect late coupling PVCs at intervals >400 ms, whereas in other PVTs we should expect to see PVCs at intervals <400 ms.^1^

*Dr Bunch:* Dr Scheinman, what is your differential diagnosis for patients presenting with polymorphic wide complex tachycardia?

*Dr Scheinman:* These patients highlight the importance of careful evaluation of the clinical as well as ECG characteristics of polymorphous VT in order to fashion appropriate therapy. In analyses of such recordings (Box 1) it is important from the onset to exclude multiple apparent ECG forms due to artifact or those showing very rapid pre-excited atrial fibrillation. The latter will generally show 1 predominant morphology but may become difficult to distinguish from PVT in the presence of conduction over multiple pathways as well as aberrant conduction over the normal conduction system. Once these entities have been excluded, we then divide these arrhythmias into pleomorphic VT, which is defined as VT showing 2 or monomorphic patterns.^2^ The latter is usually associated with coronary artery disease or cardiomyopathy and should be readily distinguished from those showing the beat-to-beat changes in QRS forms seen in PVT. In this exercise, we will focus on the patients who present with PVT patterns.

Box 1 **Electrocardiographic analyses of multiform ventricular tachycardia**

Step 1: Exclude artifact or pre-excited atrial fibrillation
Step 2: Pleomorphic ventricular tachycardia (VT) 2 or more monomorphic patterns
Step 3: Polymorphic VT (beat-to-beat change in QRS amplitude and morphology)
Step 4: True torsades QTc >500 ms and premature ventricular complex (PVC) coupling interval >400 ms
Step 5: Pseudo-torsades QTc <500 ms and PVC coupling interval <400 ms

*Dr Singh:* Dr Scheinman, what are the clinical implications of classic TdP vs pseudo-TdP?

*Dr Scheinman:* Classic torsades (from the French “turning of the points”)^2^ is characterized by marked prolongation of the QTc (>500 ms) as well as a relatively long coupling interval between the preceding QRS and initiating PVC (>400 ms).^3^ Clinically, the setting is most often seen in those with congenital or acquired LQTS. In the congenital LQTS the usual genetic abnormality results in loss of function in the potassium current, giving rise to prolongation of the QTc. Less commonly, the prolonged QT can result from a gain of function in sodium or calcium currents. Alternatively, this picture may result from severe loss of electrolytes, exposure to QT-prolonging drugs, or severe bradycardia. The acquired LQTS is almost always associated with situations resulting in loss of the IKr current. This is the picture presented by patient 1, accompanied by the markedly prolonged QT as well as the relatively long coupling interval. In this patient, although genetic studies were unremarkable, it is important to remember that 15% of patients with LQTS will not have a pathogenic mutation. Out of an abundance of caution she is treated with nadolol and instructed regarding management of gastrointestinal losses.

An entity described as pseudo-TdP was recently introduced by Viskin and colleagues^4^ and is defined as a situation where there may be mild prolongation of the QTc related to either idiopathic VT or underlying cardiac issues such as ischemic cardiac disease and/or exposure to drugs that prolong the QT interval, and the patient presents with PVT, which is in fact unrelated to the QT lengthening. The second patient in this report fits into this category, presenting with evidence of ischemia as well as exposure to 2 QT-prolonging drugs and whose arrhythmia responded to revascularization. Both cases nicely demonstrate the importance of making the distinction between true and pseudo-TdP in terms of therapeutic interventions. In a study of 190 patients with PVT, the QT intervals in patients with pseudo-TdP were significantly shorter than patients with true TdP (QTc 491.4 ± 25.2 ms vs 564.6 ± 75.6 ms, *P* < .001).^1^

*Dr Bunch:* Dr Scheinman, in both these examples the QTc was abnormal and prolonged. In patients without QTc prolongation and PVT, is the evaluation and management different?

*Dr Scheinman:* The other consideration in the evaluation of PVT is the patient who presents with no structural cardiac disease and normal or only slightly prolonged QTc and PVT—the so-called short-coupled TdP originally described by Leenhardt and colleagues,^5^ in which the coupling interval of the triggering PVC was very short (between 200 and 400 ms), as opposed to that found in true TdP, which is generally >400 ms. In addition, it is appreciated that the triggering PVC may arise from Purkinje tissue (His-Purkinje system) as well as from myocardial sites of origin—for example, the right ventricular outflow tract, papillary muscles, or moderator band. A number of recent studies have focused on genetic mutations associated with increased discharge from the His-Purkinje system, including mutations in DPP6 producing gain of function of Ito or syndromes associated with gain in function of SCN5A channels (multifocal ectopic Purkinje-related premature contractions).^6^ Others are associated with abnormal discharge from myocardial cells. For example, a new syndrome associated with ventricular fibrillation involves loss of function of RYR2 channels.^7^ It is very important to distinguish short-coupled TdP from other forms of PVT, since therapy involves use of quinidine^8^ or catheter ablation of Purkinje or myocardial triggers.^9^

**Figure 5:**
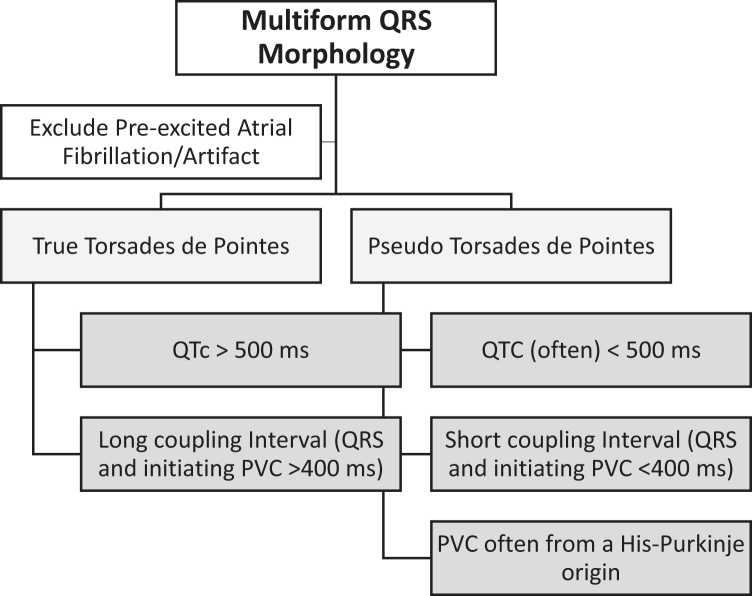
Process for arriving at a proper diagnosis and treatment strategy. PVC = premature ventricular complex.

In conclusion, careful integration of the clinical picture as well as important features of the presenting ECGs, as shown in the summary in Figure 5, are important at arriving at a proper diagnosis and treatment strategy.
